# Pesticides: Toxic Legacy

**DOI:** 10.1289/ehp.115-a190b

**Published:** 2007-04

**Authors:** Julia R. Barrett

An ongoing prospective cohort study in New York City reveals for the first time that prenatal exposure to the pesticide chlorpyrifos damages children’s neurodevelopment with negative impacts on cognition, motor skills, and possibly behavior. These findings, published in the December 2006 *Pediatrics*, mirror animal studies of the chemical, which the EPA banned for residential use in 2001. Concern persists, though, because children exposed prior to the ban may experience lifelong consequences, and population exposure continues through nonresidential uses.

The cohort study, begun in 1997, focuses on prenatal exposure to ambient and indoor pollutants and effects on neurocognitive development and other end points. The study population comprises inner-city minority women recruited during pregnancy and their children born between February 1998 and May 2002; data collected include biological samples, exposure assessments, maternal interviews, and developmental testing of the children. Of 254 children who had reached their third birthday, those with the highest prenatal chlorpyrifos exposure had significantly lower scores on mental and motor indices and more problems associated with attention deficits, hyperactivity, and pervasive developmental disorders. Effects were most marked in motor development.

“I’m not surprised that they showed the motor effects as more robust, because at an early age that’s mostly what one sees,” says Edward Levin, a professor of psychiatry and psychological and brain sciences at Duke University. Development of language and other cognitive skills comes later, as does the ability to control behavior. “It actually follows in very well with the preclinical work identifying the developmental neurotoxicity of chlorpyrifos,” Levin says.

Much of that work was conducted by Theodore Slotkin, a professor of pharmacology and cancer biology at Duke, who characterizes the current study as landmark. “There’s a large underpinning of animal research for organophosphate pesticides, and particularly for chlorpyrifos, that points to bad outcomes in terms of effects on brain development and behavior,” he says. Extrapolating results from animal studies to human health can be difficult, but this study pinpointed exposures and controlled for numerous variables that generally confound epidemiologic study. Further, he says, the study confirms that all the animal findings that led to the decision to ban use in the home turned out to be true.

“In animal studies [chlorpyrifos-induced] behavioral effects are not reversible. We don’t know in children whether the kind of attention problems that appear to be associated with chlorpyrifos exposure are treatable,” says lead author Virginia Rauh, an associate professor of clinical population and family health at Columbia University. The researchers will follow the children until they are 10 to 11 years old and possibly longer. “It’s important to continue,” says Rauh. “The [current] ban is likely not sufficient. We don’t know that chlorpyrifos is safe at any level.”

## Figures and Tables

**Figure:**
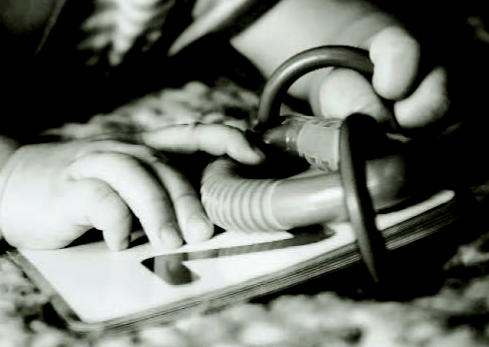
Not over yet Although chlorpyrifos was banned for household use in 2001, today’s children still face the threat of neurotoxic effects.

